# Transition Metal Slab Gliding: One Key Process for Activating Anionic Redox Reaction in P2‐Type Transition Metal Oxide Cathodes

**DOI:** 10.1002/advs.202501852

**Published:** 2025-04-03

**Authors:** Dongxiao Wang, Feihu Zou, Weiguang Lin, Xiaochen Zhang, Xinran Zhang, Xingguo Qi, Shuyin Xu, Huican Mao, Dongdong Xiao, Shigang Lu, Bingkun Guo, Yong‐Sheng Hu, Yingchun Lyu

**Affiliations:** ^1^ Materials Genome Institute Shanghai University Shanghai 200444 China; ^2^ Beijing National Laboratory for Condensed Matter Physics Institute of Physics Chinese Academy of Sciences Beijing 100190 China; ^3^ HiNa Battery Technology Co., Ltd. Liyang 213300 China; ^4^ School of Physical Science and Technology Inner Mongolia University Hohhot 010021 China; ^5^ Department of Energy Storage Science and Engineering School of Metallurgical and Ecological Engineering University of Science and Technology Beijing Beijing 100083 China; ^6^ College of Sciences and Institute for Sustainable Energy Shanghai University Shanghai 200444 China

**Keywords:** anionic redox reaction, ion migration, P2‐type cathode, sodium‐ion battery, transition metal slab gliding

## Abstract

Anionic redox chemistry is crucial for determining the capacity and stability of layered oxide cathodes for Na‐ion batteries. However, the driving forces of anionic redox remain elusive. Na–O–A (A = alkali metal, alkaline earth metal, or vacancy) configurations have been identified as key to activating anionic redox reactions in transition metal (TM)‐deficient oxide materials. However, reversible oxygen redox reactions are also observed in TM‐stoichiometric oxide materials, although the Na–O–A configurations cannot be established in these systems. A TM‐stoichiometric P2‐type Na_2/3_Cu_1/3_Mn_2/3_O_2_ cathode and its Ti^4+^‐substituted analogue (Na_2/3_Cu_1/3_Mn_1/2_Ti_1/6_O_2_) are herein studied for deciphering the underlying mechanism. The incorporation of Ti^4+^ disrupts the ordered arrangement of TM layers, accelerates TM slab gliding, and facilitates TM migration, thus affording new Na–O–vacancy configurations and activating the reversible oxygen redox reaction. Consequently, Na_2/3_Cu_1/3_Mn_1/2_Ti_1/6_O_2_ exhibits an initial discharge capacity of 153 mAh g^−1^ and a capacity retention of 80% after 300 cycles at 2 C rate. This work provides plausible routes for reversible anionic redox reactions and increasing the energy density of SIBs.

## Graphical abstract

Transition metal (TM) slab gliding is a key process for activating anionic redox reaction in P‐type layered oxide materials. Disordered TM layer weakens interlayer interactions and facilitates TM slab gliding. The resulting O‐type layer promotes reversible TM migration, provides the necessary Na–O–□_TM_ configuration, and activates anionic redox reaction.

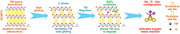

## 1 Introduction

Owing to the abundance and low cost of the corresponding resources, Na‐ion batteries (SIBs) are proposed as a promising alternative to lithium‐ion batteries for meeting the growing demand for electric vehicles and smart grids.^[^ ^1^, ^2^ ^]^ SIBs feature physicochemical properties similar to those of lithium‐ion batteries but have not been commercialized, mainly because of their low energy density. Anionic redox chemistry, as a supplement to cation redox chemistry, plays an important role in determining the capacity and energy density of layered oxide cathodes.^[^ ^3^, ^4^ ^]^ Anionic redox reaction activity was first observed in Li‐rich cathode materials, which feature Li–O–Li configurations creating nonbonding O‐2p states.^[^ ^5^ ^]^ This concept has been expanded to Na–O–A configurations (A = alkali metal, alkaline earth metal, or vacancy) in transition metal (TM)‐deficient layered oxide cathodes.^[^ ^6^, ^7^, ^8^, ^9^ ^]^

Exceptions to the Na–O–A configuration are also notable, particularly in TM‐stoichiometric layered oxide materials. Cao et al. showed that the out‐of‐plane migration of ions from the TM layer to the alkali metal layer transforms the Na–O–M_TM_ configuration into a Na–O–□_TM_ or □_Na_–O–□_TM_ configuration, where □ indicates a vacancy.^[^ ^10^ ^]^ This behavior explains the oxygen redox chemistry in O‐type cathodes (where Na ions occupy octahedral sites), as TM ions can easily migrate from the TM slab to the Na slab through an octahedral–tetrahedral–octahedral path.^[^ ^11^, ^12^ ^]^ Some TM‐stoichiometric P‐type oxide materials (where Na ions occupy prismatic sites), such as P2‐Na_2/3_Cu_1/3_Mn_2/3_O_2_, do not show oxygen redox reactions.^[^ ^13^, ^14^ ^]^ However, anionic redox reactions are also observed in certain TM‐stoichiometric P‐type oxide materials, such as Na*_x_* M*_y_* Mn_1_ *_−y_* O_2_ (M = Ni, Co, Fe, Ru, Mo, etc.).^[^ ^15^, ^16^, ^17^, ^18^, ^19^, ^20^ ^]^ Voronina et al. reported that Na_0.6_Co_0.78_Ru_0.22_O_2_ experiences oxygen redox reactions activated by a P2‐to‐OP4 phase transition during desodiation,^[^ ^21^ ^]^ unlike P2‐type Na_0.67_CoO_2_, which shows no oxygen redox reactions and phase transition. Zhang et al. found that the substitution of Mo for Mn in NaMnO_2_ effectively suppresses phase transitions while inhibiting oxygen oxidation.^[^ ^22^ ^]^ These findings suggest a strong relationship between the oxygen redox reaction activity and phase transitions, although the underlying driving forces, particularly in P2‐type materials without Na–O–A configurations, remain unclear.

Herein, we demonstrate a TM‐stoichiometric P2‐type Na_2/3_Cu_1/3_Mn_2/3_O_2_ cathode and its Ti^4+^‐substituted analogue (Na_2/3_Cu_1/3_Mn_1/2_Ti_1/6_O_2_) and reveal that the incorporation of Ti^4+^ disrupts the ordered arrangement of TM layers and thus accelerates TM slab gliding and facilitates TM migration. These processes afford new Na–O–vacancy configurations to activate the reversible oxygen redox reaction and increase the reversible capacity and energy density. Consequently, the NCMTO cathode shows a high cycling stability and capacity retention of 80% after 300 cycles at a 2 C rate. The corresponding full cell coupled with hard carbon shows an excellent cycling performance and a high initial coulombic efficiency of ≈98%. TM slab gliding is suggested to be a key process for the oxygen redox reaction activity in P2‐type cathode materials.

## 2 Results and Discussion

### 2.1 Material Characterization

**Figure 1:**
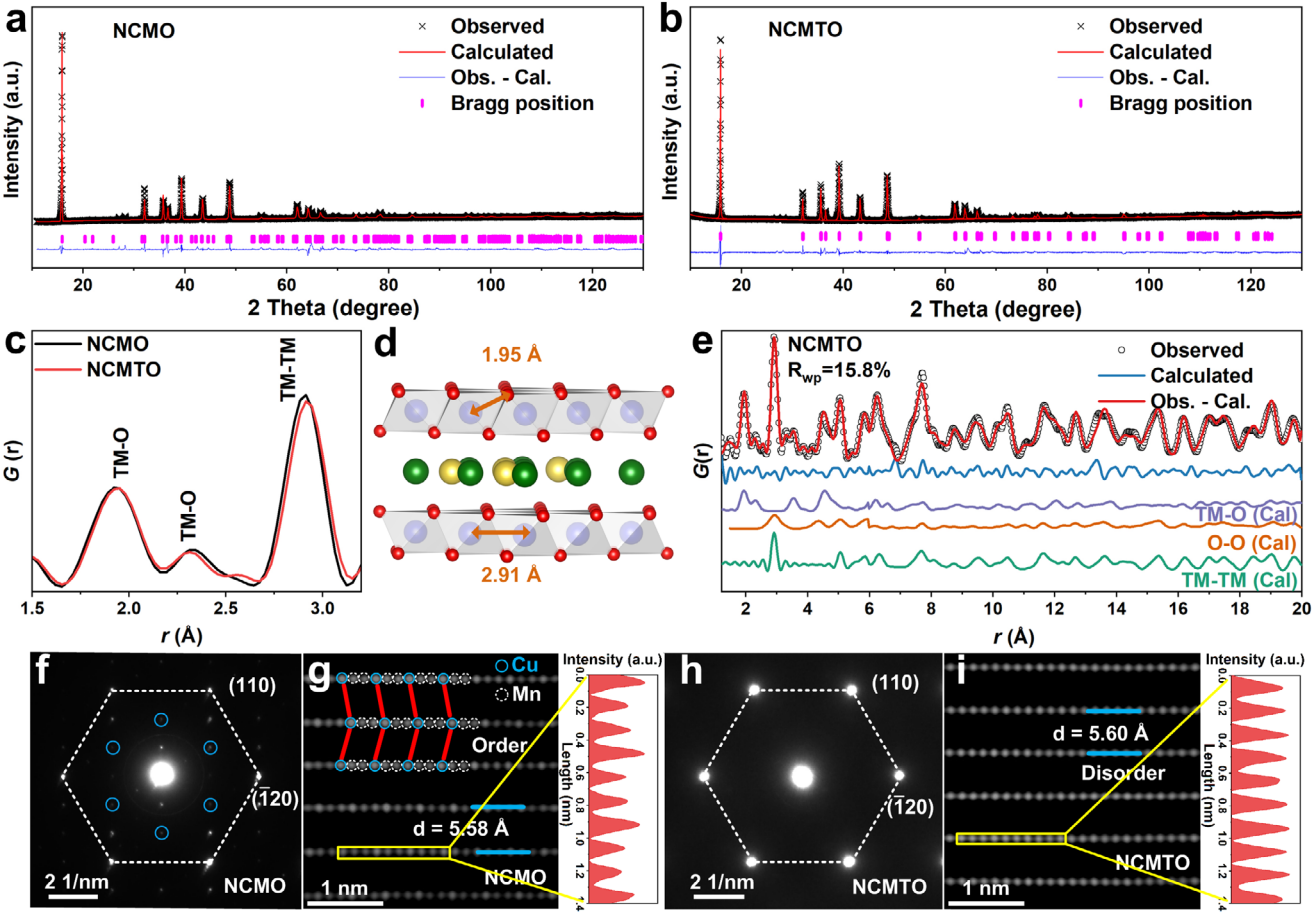
Structural characterizations. Rietveld refinements of the XRD patterns of a) Na_2/3_Cu_1/3_Mn_2/3_O_2_ (NCMO) obtained using the *P*6_3_ space group and b) Na_2/3_Cu_1/3_Mn_1/2_Ti_1/6_O_2_ (NCMTO) obtained using the *P*6_3_ */mmc* space group. c) Short‐range pair distribution function (PDF) profiles of NCMO and NCMTO. d) Schematic diagram of the TM─O and TM─TM bond in NCMO and NCMTO. e) PDF fit of NCMTO obtained using the *P*6_3_ */mmc* space group. Selected area electron diffraction patterns in the [001] hex zone axes of f) NCMO and h) NCMTO. Atomic‐resolution HAADF‐STEM images of g) NCMO and i) NCMTO were acquired along the [120] direction.

A series of Na_2/3_Cu_1/3_Mn_2/3‐x_Ti_x_O_2_ (x = 0, 1/12, 1/6, and 1/4) composites were synthesized via a simple and scalable solid‐state reaction, and all the samples demonstrated a hexagonal P2 structure (Figure S1, Supporting Information). The sample with x = 1/6 (namely NCMTO) was mainly investigated by comparing it with Na_2/3_Cu_1/3_Mn_2/3_O_2_ (NCMO) in this study. The elemental contents of NCMO and NCMTO were confirmed by inductively coupled plasma‐atomic emission spectrometry (Table S1, Supporting Information). The *P*6_3_ space group was used for Rietveld refinement of the X‐ray diffraction (XRD) patterns of both samples (**Figure** **1**a; Figure S2, Supporting Information),^[^ ^23^ ^]^ with the related details provided in Tables S2 and S3 (Supporting Information). The refinement results show that Mn^4+^ occupies the 4d site and Cu^2+^ occupies the 2b site with an ordered honeycomb structure for NCMO. However, in NCMTO, the ordered honeycomb structure was less pronounced, possibly because Ti^4+^ had an ionic radius (*R*_CN = 6_ = 0.605 Å) lying between those of Cu^2+^ (*R*_CN = 6_ = 0.72 Å) and Mn^4+^ (*R*_CN = 6_ = 0.53 Å) and thus effectively mitigated the size difference between the ions within the TM slabs. The Rietveld refinement of NCMTO based on the TM‐disordered *P*6_3_ */mmc* space group (Figure 1b; Table S4, Supporting Information) resulted in a smaller *R*_wp_ value (3.52%). The local structures of NCMO and NCMTO were examined using pair distribution function (PDF) analysis (Figure 1c; Figure S3, Supporting Information). The similarity between the PDF profiles of NCMO and NCMTO suggested that Ti^4+^ substitution did not alter the overall structure. The intensity loss of TM‐related peaks upon the incorporation of Ti^4+^ was attributed to the concomitant decrease in the X‐ray scattering factor. Owing to the Jahn–Teller distortion of Cu^2+^ (d^9^), the six TM─O bonds in an octahedral setting were split into two long (2.34 Å) and four short (1.93 Å) bonds.^[^ ^15^ ^]^ The incorporation of Ti^4+^ decreased the difference between these bond lengths, suggesting the suppressed Jahn–Teller distortion (Figure 1d). A fine PDF fit was obtained for NCMTO (*R*_wp_ = 15.8%) using the *P*6_3_ */mmc* space group (Figure 1e).

The scanning electron microscopy (SEM) images of NCMO and NCMTO (Figure S4, Supporting Information) showed a well‐crystallized structure with a particle size of 2–3 µm. The transmission electron microscopy (TEM) imaging of NCMTO revealed the presence of plate‐like microcrystals (Figure S5, Supporting Information), while high‐resolution TEM imaging revealed lattice fringes with an interlayer spacing of 5.6 Å, which well matched the (002) planes of the P2‐type structure. The results of elemental mapping indicated the uniform distributions of Na, Cu, Mn, Ti, and O. The TM arrangement was further examined using selected area electron diffraction (Figure 1f,h), with the hexagonal spot arrangement confirming the P2 structure of both samples. The weak spots observed between the bright spots for NCMO (Figure 1f) indicated the presence of TM ordering.^[^ ^24^ ^]^ However, such spots were not observed for NCMTO (Figure 1h), which suggested that the incorporation of Ti^4+^ disrupted the ordered arrangement of the TM layers. High‐angle annular dark‐field scanning transmission electron microscopy (HAADF‐STEM) imaging (Figure 1g) revealed local Cu–Mn–Mn–Cu ordering within the TM layers aligned along the [120] direction in NCMO. The *Z*‐contrast observed for Cu was notably higher than that observed for Mn because of the higher atomic number of the former (*Z* = 29 for Cu vs *Z* = 25 for Mn). In stark contrast, the HAADF‐STEM imaging of NCMTO (Figure 1i) revealed a disordered TM layer, indicating the uniform and random distribution of all elements beneath the projected atomic columns. Line profile analysis provided further evidence for the disrupted Cu/Mn/Ti arrangement within the TM layer, as each atomic column exhibited the same intensity.

### 2.2 Electrochemical Characterization

**Figure 2:**
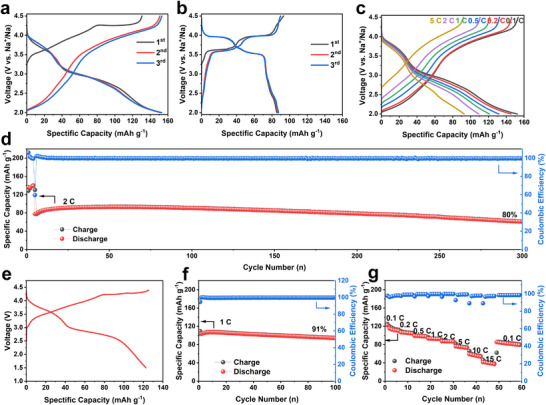
Electrochemical properties. Charge–discharge curves of a) NCMTO and b) NCMO at 0.1 C in half‐cells for the first three cycles. c) Rate performance of NCMTO. d) Cycling performance of NCMTO at 2 C. e) Initial charge–discharge curves at 0.1 C, f) cycling performance at 1 C, and g) rate performance of NCMTO//hard carbon full‐cells.

The electrochemical behaviors of NCMO and NCMTO were examined in half‐ and full‐cells. **Figures** **2**a,b, and S6 (Supporting Information) show the charge–discharge curves of the samples, between 2.0 and 4.5 V versus Na^+^/Na for the first three cycles at 0.1 C (1 C = 130 mA g^−1^). The charge and discharge capacities of NCMO (94 and 84 mAh g^−1^, respectively) minimally exceeded the theoretical capacity considering the Cu^2+^/Cu^3+^ redox reaction with the intercalation of 0.33 Na^+^ per formula unit. However, this extra capacity could not be reversed during discharge and may result from the decomposition of the electrolyte and/or residual surface alkali at high voltages.^[^ ^25^, ^26^, ^27^ ^]^ The two plateaus at 3.6 and 4.0 V were attributed to the Cu^2+^/Mn^4+^ charge ordering–induced structural transformations.^[^ ^28^, ^29^, ^30^ ^]^ The charge–discharge profiles of NCMTO featured no such plateaus and had a characteristic sloping‐curve shape indicative of a solid‐solution reaction. NCMTO showed a first charge capacity of 130 mAh g^−1^, corresponding to the extraction of 0.5 Na^+^ per formula unit (0.33 Na^+^ per formula unit from the Cu^2+^/Cu^3+^ redox reaction and 0.17 Na^+^ per formula unit from the oxygen redox reaction). The first discharge capacity of NCMTO (153 mAh g^−1^) corresponded to the intercalation of 0.6 Na^+^ per formula unit. The insertion of additional Na^+^ during discharge indicated the participation in the reduction of Mn^4+^ at low voltages. In situ differential electrochemical mass spectrometry (DEMS) was performed to investigate whether oxygen release occurs during the charging/discharging processes, as illustrated in Figure S7 (Supporting Information). No signals for O_2_ or CO were observed during the measurement, while a small amount of CO_2_ was detected for both samples beyond 4.3 V. The release of CO_2_ may have resulted from the unstable decomposition of electrolytes in addition to the decomposition of Na_2_CO_3_ on the surface.^[^ ^31^, ^32^ ^]^ Since no O_2_ product is detected, the O in CO_2_ cannot be related to the lattice oxygen. It suggests the reversibility of oxygen redox in NCMTO.^[^ ^33^ ^]^ NCMTO exhibited reversible capacities of 153, 145, 132, 121, 110, and 96 mAh g^−1^ at current rates of 0.1, 0.2, 0.5, 1, 2, and 5 C, respectively (Figure 2c). When the current rate was decreased from 5 to 0.1 C, a capacity of 145 mAh g^−1^ was recovered, corresponding to ∼95% of the pristine capacity at 0.1 C and indicating a high structural stability. The galvanostatic intermittent titration technique (GITT) was used to determine Na diffusion coefficients (Figures S8 and S9, Supporting Information). The apparent Na diffusion coefficient of NCMTO ranged from 6.51 × 10^−12^ to 1.19 × 10^−8^ cm s^−1^ and was comparable with that of NCMO at low voltages (Figure S10, Supporting Information). When the voltage exceeded 4.3 V, the diffusion coefficient of NCMTO decreased, possibly because of the low oxygen redox reactions kinetics, which is often observed in layered cathodes.^[^ ^34^ ^]^ During long‐term cycling performance testing at 2 C (Figure 2d), NCMTO demonstrated a capacity retention of 80% after 300 cycles.

Hard carbon was used as an anode material to assemble full cells, with its electrochemical and cycling properties presented in Figure S11 (Supporting Information). The NCMTO cathode was coupled with the hard carbon anode by adjusting the N/P ratio to 1.02:1. The anode was presodiated in a Na‐metal half‐cell for one cycle. The first charge and discharge profiles of the full‐cell at 0.1 C (Figure 2e) had a sloping curve‐type shape and a high reversibility. The first discharge capacity was calculated as ≈123 mAh g^−1^ based on the mass of the cathode. The full cell showed an excellent cycling behavior and rate performance (Figure 2f,g), featuring a capacity retention of 91% after 100 cycles at 1 C. The specific discharge capacity at 1, 5, and 10 C were 93, 76, and 59 mAh g^−1^, respectively. Even at a high rate of 15 C (4 min discharge), the reversible capacity was 47 mAh g^−1^ (≈41% of the initial capacity at 0.1 C). The rate performance in the full‐cell test surpassed that in the half‐cell test, in agreement with previous reports.^[^ ^35^, ^36^ ^]^ The half‐cells and full‐cells cyclic performance of NCMO is shown in Figures S12 and S13 (Supporting Information). NCMO and NCMTO both show high cyclic stability. It is further proved that even when high–voltage oxygen redox is activated, NCMTO still has excellent cycle stability and a high reversible capacity.

### 2.3 Charge Compensation

**Figure 3:**
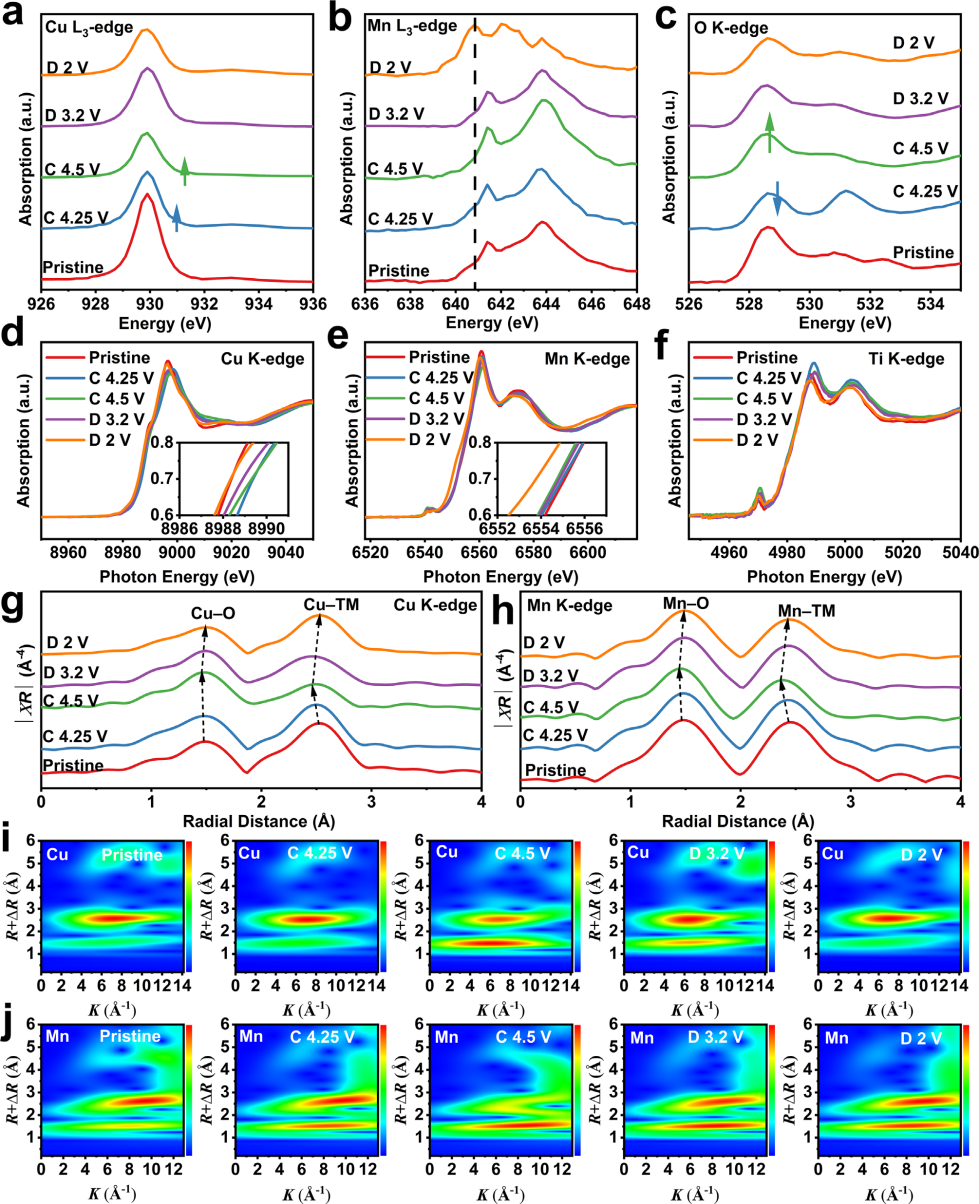
Electronic structure evolution of NCMTO. a) O K‐edge, b) Cu L_3_‐edge, and c) Mn L_3_‐edge ex situ soft X‐ray absorption spectra were recorded in the total electron yield mode at various charge and discharge states during the first cycle. d) Cu K‐edge, e) Mn K‐edge, and f) Ti K‐edge ex situ hard X‐ray absorption spectra were recorded at various charge and discharge states during the first cycle. *R*‐space plots derived from ex situ g) Cu K‐edge and h) Mn K‐edge extended X‐ray absorption fine structure (EXAFS) spectra. Contour plots obtained by the wavelet transform analysis of the ex situ i) Cu K‐edge and j) Mn K‐edge EXAFS spectra.

The charge compensation mechanism of NCMTO was investigated by synchrotron X‐ray absorption spectroscopy (XAS) measurements for different charge and discharge states during the initial cycle. **Figure** **3**a shows the Cu L_3_‐edge spectra of NCMTO. During charge–discharge, the position of the peak centered ≈930 eV remained stable, while a distinct shoulder peak minimally above 932 eV emerged at 4.25 V and persisted at 4.5 V. This peak was associated with the hybridization of Cu^2+^ with the ligand oxygen and formation of oxygen hole states, indicating the oxidation of Cu^2+^.^[^ ^37^, ^38^ ^]^ During the subsequent discharge, this peak disappeared, suggesting the reduction of Cu^3+^ to Cu^2+^. The corresponding Mn L_3_‐edge spectra (Figure 3b) showed peaks at 641.4 and 643.8 eV during charging, which confirmed the inactiveness of Mn^4+^. While emergence of Mn^3+^ ions, characterized by a peak at 640.8 eV, is observed upon discharging to 2.0 V. Thus, the partial reduction of Mn^4+^ to Mn^3+^ provided the additional capacity observed during discharge. Figure 3c shows the corresponding O K‐edge spectra, with the area under the pre‐edge peaks providing information on holes and the average effective charge of the O 2p–TM 3d hybridized orbitals.^[^ ^6^ ^]^ The notable reduction in the intensity of the O K‐edge pre‐edge peaks upon charging to 4.25 V aligns with the characteristics reported for the Zhang–Rice (ZR) singlet state in high‐*T*_c_ superconducting cuprates.^[^ ^38^, ^39^ ^]^ The ZR state refers to a correlated electronic state formed between a Cu^2+^ ion with one hole in its e_g_ orbital and a ligand O with one hole in its p orbital (Cu^2+^: 3d^9^–O: 2p^5^, or d^9^ L, where L denotes a ligand hole).^[^ ^40^, ^41^ ^]^ At 4.5 V, the O K‐edge pre‐edge peak considerably intensified, which suggested that a significant portion of the holes compensating for the deintercalation of Na‐ions reside primarily in O 2p states. This result demonstrates the pivotal role played by lattice oxygen in the subsequent redox reaction.^[^ ^42^ ^]^ Figure S14 (Supporting Information) shows the X‐ray photoelectron spectroscopy analysis performed on NCMTO. The oxygen oxidation observed at high voltages confirmed the occurrence of oxygen redox reactions in NCMTO.

Figure 3d–f depicts the normalized Cu, Mn, and Ti K‐edge X‐ray absorption near‐edge structure (XANES) spectra of NCMTO across various charge and discharge states. Upon charging to 4.25 V, the Cu K‐edge shifts to higher energy, which confirms the oxidation of Cu^2+^ (Figure 3d). The absence of a notable shift at 4.5 V indicates that this stage involves oxygen oxidation but not TM oxidation for charge compensation. The energy shift toward the pristine state observed during the subsequent discharge indicates the reduction of Cu^3+^ to Cu^2+^, in good agreement with Cu L_3_‐edge data. The Mn K‐edge spectra (Figure 3e) remained nearly unchanged during the first charge, implying that the Mn oxidation state remained at Mn^4+^, in agreement with the Mn L_3_‐edge data. Upon discharge, the Mn K‐edge shifted to an even lower energy than that of the pristine electrode, demonstrating the reduction of Mn^4+^. The Ti K‐edge spectra (Figure 3f) remained unchanged across all measured states, suggesting the inactivity of Ti^4+^.

The local structure and bond distances of NCMTO were also revealed by Cu and Mn K‐edge extended X‐ray absorption fine structure (EXAFS) spectra (Figure 3g,h). Upon desodiation, the Cu─O bond length minimally decreased because of the oxidation of Cu and O (Figure 3g), whereas the Cu─TM bond length considerably decreased.^[^ ^43^ ^]^ Similarly, owing to the TM and O reduction upon sodiation, the Cu─O (Cu─TM) bond length increased to the pristine value. Similar trends were revealed by the analysis of the corresponding Mn K‐edge EXAFS spectra (Figure 3h), indicating the good reversibility of the coordination environment of Mn ions. To supplement the EXAFS results and more intuitively explore the changes in the coordination environments of TM ions, we performed the wavelet transform (WT) analysis of Cu K‐edge spectra (Figure 3i).^[^ ^44^ ^]^ The scattering peaks located at (6.5 Å^−1^, 1.5 Å) and (7.2 Å^−1^, 2.5 Å) in the corresponding contour plots were attributed to the contributions of Cu─O and Cu─TM features, respectively, and the peak intensities were closely related to the evolution of local coordination environments, such as TM migration. The Cu─O bond strengthening observed upon charging from 4.25 to 4.5 V indicated that oxygen oxidation enhanced the Cu─O bond strength. The Cu─TM bond weakening suggested a phenomenon similar to that observed for other layered structural materials capable of activating oxygen redox reactions, which are accompanied by the migration of TM‐layer atoms toward the Na layer.^[^ ^10^, ^45^ ^]^ Notably, the subsequent discharge resulted in an intensity recovery. The WT analysis of Mn K‐edge spectra revealed peak intensity changes similar to those observed in Cu K‐edge spectra (Figure 3j).

### 2.4 Structural Evolution

**Figure 4:**
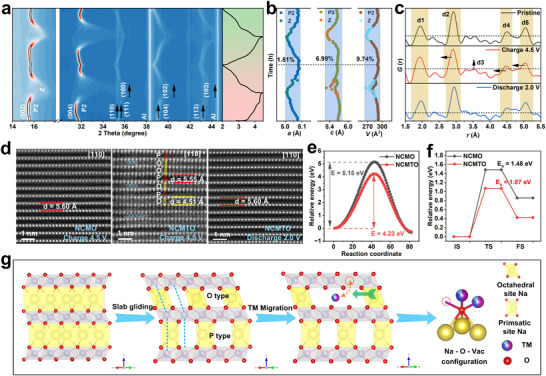
Crystal structure evolution of NCMTO. a) In situ XRD contour plot of NCMTO constructed during the first two cycles and b) evolution of lattice parameters (*a*, *c*) and unit cell volume (*V*). c) Ex situ PDF patterns of NCMTO in various charge‐discharge states (pristine, first‐charged to 4.5 V, and first‐discharged to 2.0 V). d) Atomic‐resolution HAADF‐STEM images of NCMO and NCMTO in different states (red area: O‐type stack, yellow area: P‐type stack, blue circle: migrating transition metal (TM) ions). e) Stacking fault energy profiles of NCMO and NCMTO. f) Energy barrier profiles of Cu ion diffusion in NCMO and NCMTO. g) Schematic representation of TM‐stoichiometric P‐type oxide materials forming a Na–O–□_TM_ configuration to activate oxygen redox reactions.

To explore the structural evolution of NCMTO during Na^+^ extraction and insertion, in situ XRD patterns were collected during the first two cycles at 0.1 C. Unlike NCMO, which experienced two phase transitions (P2 → P'2, P″2) during charging at 2.5–4.5 V (Figure S15, Supporting Information), NCMTO experienced no structural transformations below 4.25 V, as no new peaks were observed (**Figure** **4**a). The (002) and (004) peaks shifted to lower angles, which indicated lattice expansion along the *c*‐axis due to the electrostatic repulsion between the two adjacent oxygen layers. Compared with the two‐phase reaction, this single‐phase process showed fast Na diffusion kinetics.^[^ ^46^ ^]^ During further desodiation, NCMTO experienced TM layer slippage. The (002) and (004) peaks broadened, lost intensity, and shifted to higher angles, which indicated a P2 → Z phase transition. At the end of charging (4.5 V), pronounced Z‐phase features were observed.^[^ ^47^, ^48^ ^]^ The same changes occurred in the second cycle, demonstrating the excellent reversibility of NCMTO. The calculated lattice parameters (Figure 4b) demonstrated a change in the TM interlayer distance during the oxygen redox process. In contrast, such TM slab gliding was not observed at this voltage in NCMO, which may be the root cause of the inactivity of the oxygen redox reaction therein. X‐ray PDF analysis was performed for different charge and discharge states to gain further local structural insights (Figure 4c). The TM–O and TM–TM peaks decrease during the charging process as suggested by EXASF. The increased intensity of the peak at 3.51 Å in the charged state is associated with the P2 → Z phase transition, particularly at a high concentration of Na vacancies. During this transition, some of the TM ions migrated from the TM layer to the Na layer.^[^ ^49^ ^]^ The d3 decrease in the discharged state demonstrated the reversibility of TM migration (Figure S16, Supporting Information). The results were consistent with and could be corroborated by the XAS analysis.

To further verify TM migration, the HAADF‐STEM images of NCMTO and NCMO electrodes were examined in the initial charged and discharged states (Figure 4d). Upon charging to 4.5 V, two interlayer distances were observed, namely ≈5.55 Å (P type) and ≈4.51 Å (O type).^[^ ^50^ ^]^ It is the distinct characteristics of the Z phase, including the presence of adjacent layers that were either aligned or offset relative to each other. A fraction of TM ions (blue circles) migrated to the Na layer at 4.5 V, in line with the PDF analysis. During the charging of NCMTO, TM slab gliding allowed TM ion migration from the TM slab to the Na slab through the octahedron–tetrahedron–octahedron pathway. This migration resulted in the formation of vacancies within the TM layers, which effectively transformed the original Na–O–TM configuration into a Na–O–□_TM_ configuration, thereby activating the oxygen redox reaction.^[^ ^10^ ^]^ DFT calculation by Cao et al. shows that there are no definitive critical amounts of vacancies required for the reversibility of the oxygen redox reaction in sodium‐ion battery cathodes.^[^ ^10^ ^]^ All the oxygen atoms adjacent to the □_TM_ possess the redox ability, while the activation of oxygen redox shows a positive feedback loop to the TM ion migration and the resultant □_TM_.^[^ ^51^ ^]^ Upon discharge to 2.0 V, the NCMTO reverted to the P2 phase, and the TM ions returned to their original positions in the TM layer. Conversely, in the case of NCMO, the TM ions in the TM layer could not migrate to the prismatic Na sites, and no vacancies and Na–O–vacancy configurations were therefore formed to activate the oxygen redox reaction.

The effect of Ti^4+^ incorporation on TM slab gliding was further investigated by evaluating the stacking fault energies for NCMTO and NCMO following the removal of half of the Na^+^ ions using density functional theory (DFT) calculations. Figure S17 (Supporting Information) illustrates TM slab gliding in the two samples. Each stacking fault energy profile featured two energy minima relative to the total energy of the P2 phase (Figure 4e). The initial minimum represented the P2 phase of NCMO or NCMTO, while the second (lower) minimum indicated the increased stability of the O phase. NCMTO exhibited easier TM slab gliding than NCMO, as suggested by the corresponding energy barriers (4.25 and 5.15 meV, respectively), favoring a P‐type → O‐type stacking transition. The energy barriers for Cu ion diffusion were determined using the climbing image nudged elastic band method (Figure 4f). In NCMTO, the O‐type stacking of the Na layers facilitated the migration of Cu ions from the TM octahedral sites to the Na octahedral sites (Figure S18, Supporting Information), with the corresponding energy equaling 1.07 eV (cf. 1.48 eV for NCMO). The reduced energy barrier for the TM ion migration in the structure promotes the beneficial □_TM_ and the resultant oxygen redox activity.^[^ ^10^ ^]^ It is evident that TM slab gliding activates oxygen redox reactions in P2‐type oxide cathodes (Figure 4g), creating tetrahedral and octahedral sites within the Na layers and thus generating channels for TM ion migration. This migration transforms the original Na–O–TM configuration into a Na–O–□_TM_ configuration, effectively activating the oxygen redox reaction.

**Figure 5:**
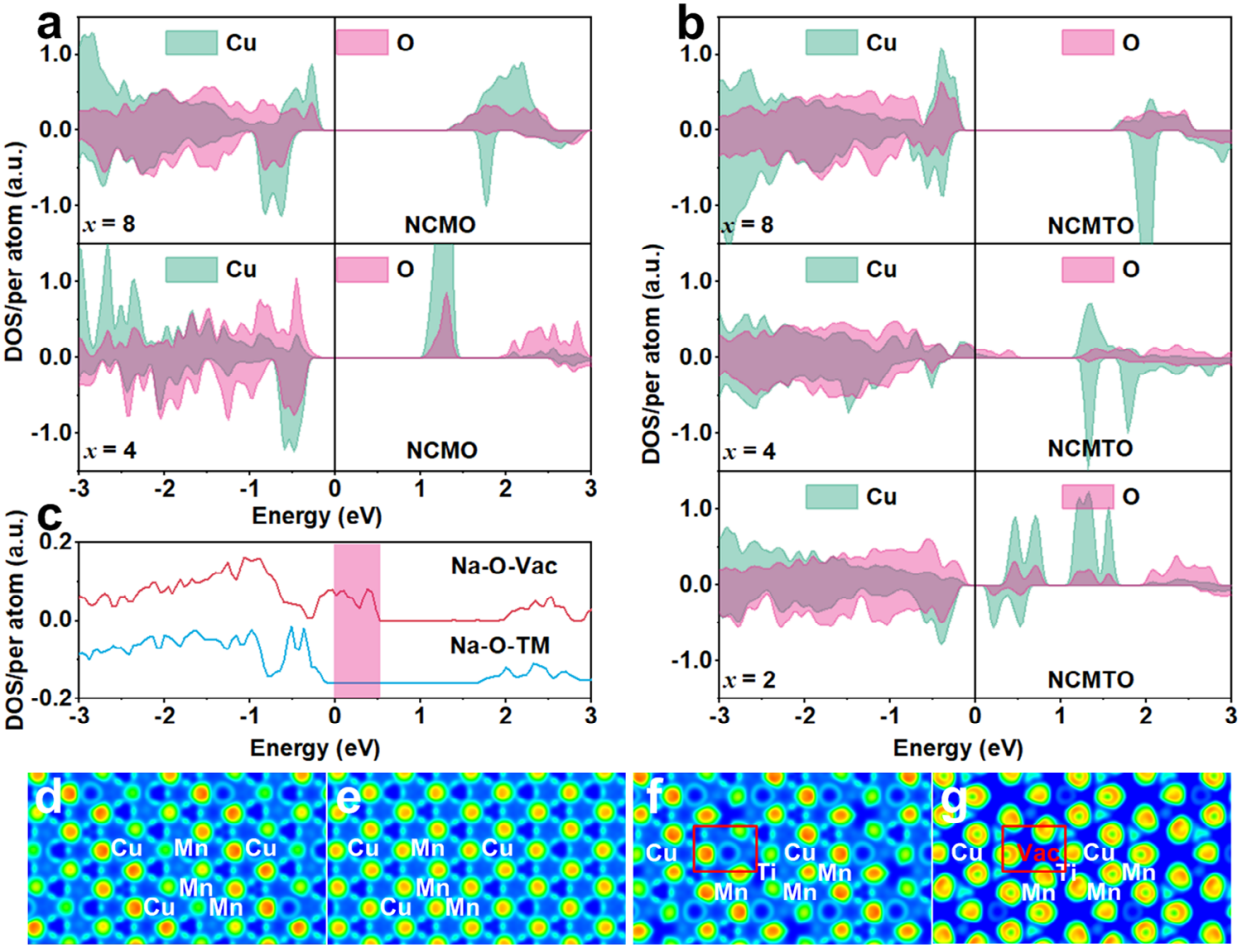
Density functional theory analysis. Projected density of states (PDOS) profiles of a) NCMO and b) NCMTO in different states. c) PDOS profile of O 2p orbitals in the local electronic and crystal environments surrounding oxygen in Na–O–□_TM_ and Na–O–TM configurations. Electron localization function images of d) NCMO and f) NCMTO and the respective half‐charged states ((e) and (g)).

To deeper understand the effect of TM slab gliding on oxygen redox reactions, the projected density of states (PDOS) and electron localization function (ELF) calculations were performed on supercells with ordered TM layers in Na*_x_* Cu_4_Mn_8_O_24_ (*x* = 8 and 4) and disordered TM layers in Na*_x_* Cu_4_Mn_6_Ti_2_O_24_ (*x* = 8, 4, and 2). The corresponding crystal structures are illustrated in Figure S19 (Supporting Information). **Figure** **5**a,b compare the PDOSs of O 2p and Cu 3d states in NCMO, NCMTO, and their half‐charged counterparts. In NCMO and NCMTO (*x* = 8), the states near the Fermi level (*E*_F_) are primarily occupied by the Cu 3d states, which suggests that charge compensation is mainly facilitated by the Cu^2+^/Cu^3+^ redox couple. As *x* is reduced to 4, signifying the extraction of half of the Na^+^ from the lattice, the upper valence bands near *E*_F_ in NCMTO become primarily occupied by O 2p states, which suggests that oxygen redox reactions take over charge compensation at this stage. In contrast, for NCMO, the O 2p orbitals are considerably more distant from *E*_F_, indicating a reduced ability to activate oxygen redox reactions. Furthermore, additional desodiation to *x* = 2 leads to a marked increase in the electron hole densities of the O 2p orbitals above *E*_F_ (0.1–0.84 eV). This suggests that oxygen redox reactions considerably contribute to capacity during deep desodiation. To probe the mechanism behind the activation of oxygen redox reactions, we conducted PDOS analysis on Na_4_Cu_3_□_1_Mn_6_Ti_2_O_24_, focusing on the O 2p states of O ions adjacent to vacancies and those near TMs (Figure 5c). The results revealed a substantial density of electron vacancies above *E*_F_ for vacancy‐adjacent O ions, whereas the density of states for O ions near TMs predominantly lied below *E*_F_. Additional PDOS analysis on the vacancy‐free TM layer of Na_4_Cu_4_Mn_6_Ti_2_O_24_ (Figure S20, Supporting Information) showed results similar to those observed for NCMO, with occupied O 2p states being distant from the *E*_F_, thus indicating that Ti^4+^ doping by itself is not sufficient for activating oxygen redox reactions. The activation of these reactions was attributed to the creation of Na–O–□_TM_ configurations, which highlights the importance of structural arrangement for facilitating these reactions.

ELF calculations were used to analyze electron distributions within NCMO and NCMTO (Figure 5d–g). In pristine NCMO and NCMTO (Figure 5d,f), electrons were evenly distributed around TM─O bonds. Upon the extraction of half of the Na^+^, the local state of oxygen ions in NCMO remained unchanged (Figure 5e), while in NCMTO, the electrons from oxygen atoms surrounding the vacancy shifted toward it (red box in Figure 5g). This demonstrated that vacancies in TM layers can adjust electron distribution within these layers. The local Na–O–□_TM_ configuration enhanced the electrochemical activity of oxygen and effectively initiated oxygen redox reactions in NCMTO. The results of the DFT analysis shed light on the role of TM slab gliding in activating the oxygen redox reactions, aligning with previous STEM analysis results and confirming the high redox activity of oxygen ions around vacancies. Therefore, for TM‐stoichiometric P‐type oxide materials, the main reason for oxygen redox activation was identified as TM slab gliding, which enabled the migration of TM ions to the Na layers and resulted in the formation of the Na–O–□_TM_ configuration.

The disorder in the TM layer weakens interlayer interactions and thus facilitates TM slab gliding,^[^ ^52^ ^]^ which is considered a key process for the activation of oxygen redox reactions in TM‐stoichiometric P‐type oxide materials. The resulting O‐type layer facilitates reversible TM migration and provides the necessary Na–O–□_TM_ configuration, which leads to enhanced oxygen redox activity. Recent studies on TM‐stoichiometric P‐type oxide materials have shown that they experience TM slab gliding during oxygen redox reactions (Table S5, Supporting Information). The absence of microcracking in NCMTO particles after 50 and 100 cycles (Figure S21, Supporting Information) suggested the reversibility of lattice gliding, as observed previously even after long‐term cycling.^[^ ^53^ ^]^ Lu et al. suggested that gliding in LiCoO_2_ contributes to its structural stability,^[^ ^54^ ^]^ with the collective gliding of CoO_6_ slabs reflecting the linkage of adjacent CoO_6_ slabs.

## 3 Conclusion

Herein, we examined the driving forces of oxygen redox reaction activity in TM‐stoichiometric P‐type oxide materials. NCMTO exhibited considerable electrochemical oxygen redox reaction activity, unlike NCMO. The results of experimental and theoretical studies suggested that this difference originated from the kinetically favorable interlayer gliding in layered TM oxides. Interlayer gliding produces octahedral sites within the Na layers, allowing TM ions to migrate from the TM slab to the Na slab through the octahedron–tetrahedron–octahedron pathway, and results in the formation of vacancies within the TM layers. This process generates new Na–O–□_TM_ configurations, activates the reversible oxygen redox reactions and boosts reversible capacity and energy density. So, TM slab gliding is suggested as a key process for activating oxygen redox reactions in TM‐stoichiometric P‐type oxide materials. Thus, our work suggests plausible routes for activating anionic redox reactions and thus provides guidelines for increasing the energy density of SIBs.

## Conflict of Interest

The authors declare no conflict of interest.

## Supporting information



Supporting Information

## Data Availability

The data that support the findings of this study are available from the corresponding author upon reasonable request.
