# Supplementary material for: Transition Metal Slab Gliding: One Key Process for Activating Anionic Redox Reaction in P2‐Type Transition Metal Oxide Cathodes
Source: Adv Sci (Weinh). 2025 Apr 3;12(26):2501852. doi: 10.1002/advs.202501852 (PMC12245024; doi:10.1002/advs.202501852)
Supplement: Supplementary file 1 — Supporting Information [file ADVS-12-2501852-s001.docx]

Supporting Information

Transition Metal Slab Gliding: One Key Process for Activating Anionic Redox Reaction in P2-Type Transition Metal Oxide Cathodes

Dongxiao Wang,^[1]^ Feihu Zou,^[1]^ Weiguang Lin,^[2]^ Xiaochen Zhang,^[1]^ Xinran Zhang,^[1]^ Xingguo Qi,^[3]^ Shuyin Xu,^[4]^ Huican Mao,*^[5]^ Dongdong Xiao,*^[2]^ Shigang Lu,^[6]^ Bingkun Guo,^[1]^ Yong-Sheng Hu,*^[2],[3]^ and Yingchun Lyu,*^[1]^

[1] D. Wang, F. Zou, X. Zhang, X. Zhang, Prof. B. Guo, Prof. Y. Lyu
Materials Genome Institute
Shanghai University
Shanghai 200444, China
E-mail: yclyu@shu.edu.cn

[2] W. Lin, Dr. D. Xiao, Prof. Y.-S. Hu
Beijing National Laboratory for Condensed Matter Physics, Institute of Physics
Chinese Academy of Sciences
Beijing 100190, China
Email: dongdongxiao@iphy.ac.cn (D. Xiao), yshu@iphy.ac.cn (Y.-S. Hu)

[3] Dr. X. Qi, Prof. Y.-S. Hu
HiNa Battery Technology Co., Ltd.
Liyang 213300, China

[4] Dr. S. Xu
School of Physical Science and Technology
Inner Mongolia University
Hohhot 010021, China

[5] Dr. H. Mao
Department of Energy Storage Science and Engineering, School of Metallurgical and Ecological Engineering
University of Science and Technology Beijing
Beijing 100083, China
E-mail: hcmao@ustb.edu.cn

[6] Prof. S. Lu
College of Sciences and Institute for Sustainable Energy
Shanghai University
Shanghai 200444, China

## Experimental Section

*Sample Preparation:* NCMO and NCMTO were prepared as follows. The stoichiometric amounts of the initial materials, including NaNO₃ (99%, Alfa Aesar), MnO₂ (99.9%, Alfa Aesar), CuO (99%, Alfa Aesar), and TiO₂ (99.9%, Alfa Aesar), were first manually mixed for 10 minutes in an agate mortar. Subsequently, the mixture was further milled in 50 ml zirconia pots using zirconia balls (Φ=5 mm) with a ball-to-powder mass ratio of 25:1 and 10 ml of ethanol, in a planetary mill (Retsch, PM100) at 500 rpm for 8 hours (10 minutes of milling followed by 2 minutes of rest). After drying, the mixture was pelletized, heated to 900 °C in air at a ramp rate of 5 °C min⁻¹, held at this temperature for 15 hours, and then cooled to room temperature. The sample was removed from the furnace, immediately transferred to an argon-filled glovebox, and stored there to avoid exposure to atmospheric moisture.

*Material Characterization:* Na, Cu, Mn, and Ti contents were determined using inductively coupled plasma atomic emission spectrometry (Agilent, 730). The samples were digested in concentrated hydrochloric acid at 200 °C. XRD patterns were recorded using a PANalytical Empyrean diffractometer equipped with a Cu Kα radiation source. Additionally, XRD Rietveld refinement was conducted using the GSAS II software to analyze the crystal structures. PDF data were acquired using a PANalytical Empyrean instrument equipped with a Ag *K*_α_ radiation source. Sample morphology and composition were examined using scanning electron microscopy (Hitachi, SU8230) coupled with energy-dispersive X-ray spectrometry. Conventional high-resolution TEM measurements were performed using a transmission electron microscope (JEOL, JEM-2100F), and HAADF-STEM images were collected using an aberration-corrected electron microscope (JEOL, ARM200F). The specimens were prepared by crushing the crystals by ultrasonic vibration in dimethyl carbonate (99%, Aladdin) and dropping them onto a carbon film supported on a copper grid. The samples were saved in an Ar-filled glove box and transferred to the TEM column using a special Gatan vacuum transfer holder to avoid any contact with the air.

*Electrochemical Measurements:* Electrochemical properties were investigated using CR2032 coin cells. Working electrodes were prepared by casting a slurry containing the active material, acetylene black, and polyvinylidene fluoride binder (70:20:10, w/w/w) and *N*-methyl-2-pyrrolidone (solvent) onto an aluminum current collector. The coin cells were assembled in an argon-filled glovebox and comprised the cathode (dried at 100 °C under vacuum for 10 h), a pure Na anode, a Whatman glass fiber separator, and the electrolyte (1 m NaClO_4_ in polycarbonate/ethylene carbonate/fluoroethylene carbonate (PC/EC/FEC, 7.5:47.5:5, v/v/v). Galvanostatic charge–discharge tests were carried out using a LANHE CT2001A battery test system in the voltage ranges of 2–4.25 and 2–4.5 V at a rate of 0.1 C. The electrode rate performance was assessed using a MACCOR 4200 battery test system. For galvanostatic intermittent titration, the cells were charged at 0.05 C for 0.5 h and then subjected to open-circuit relaxation for 4 h.

*Ex Situ Synchrotron XAS Measurements:* The electronic states of O, Mn, Cu and Ti during charge–discharge was probed by XAS. The cycled electrodes were sealed with adhesive tape in an argon-filled glovebox to prevent exposure to air. Soft XAS spectra were recorded at the BL08U1A line station of the Shanghai Synchrotron Radiation Facility (SSRF). O K-edge and Cu and Mn L-edge spectra were recorded in total electron yield modes. Hard XAS spectra of Mn, Cu and Ti K-edges were recorded at the BL11B station of the SSRF. For signal processing, a linear background was subtracted from the original XAS spectra and then normalized.

*In Situ XRD Studies:* In situ XRD patterns were recorded on a PANalytical Empyrean diffractometer equipped with a Cu *K*_α_ radiation source in the 2*θ* range of 14°–60° using an in-house-designed electrochemical cell.^[1]^

*Computational Methods:* All calculations were performed using DFT with the projector augmented plane-wave method implemented in the Vienna ab initio simulation package.^[2]^ The Perdew–Burke–Ernzerhof generalized gradient approximation was used to model the exchange-correlation potential.^[3]^ Long-range van der Waals interactions were described by the DFT-D3 approach.^[4]^ The plane-wave cut-off energy was set to 500 eV. The energy criterion in the iterative solution of the Kohn–Sham equation was set to 10^−6^ eV. The *K*-mesh resolution in real space was 0.04 2π Å^−1^. All structures were relaxed until the residual forces acting on the atoms had declined to <0.03 eV Å^−1^. The energy barriers of Cu ion diffusion were calculated using the climbing image nudged elastic band method, and seven intermediate structures, including the initial and final structures, were considered. The DFT+U method was used to treat the strong on-site coulombic interaction of localized electrons, which is not correctly described by local density approximation (LDA) or generalized gradient approximation (GGA). The effective U-J values for Cu and Mn were set to 6 and 2.4 eV, respectively, according to previous works.^[5,6]^


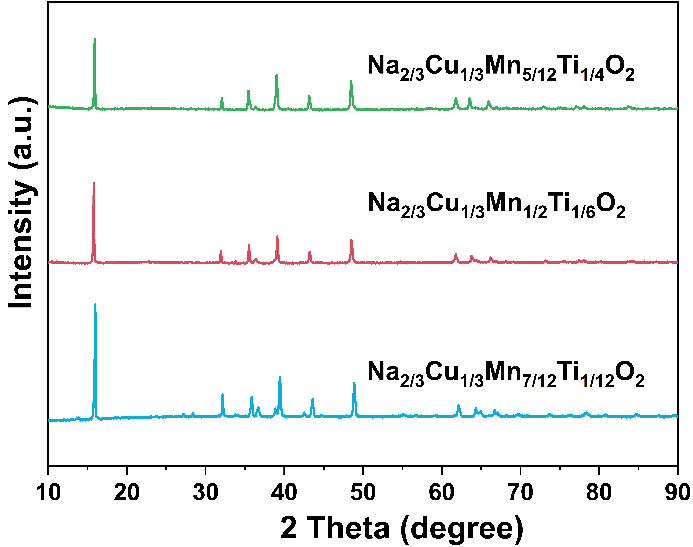


**Figure S1.** XRD patterns of different Na_0.67_Cu_0.33_Mn_0.67-x_Ti_x_O_2_.


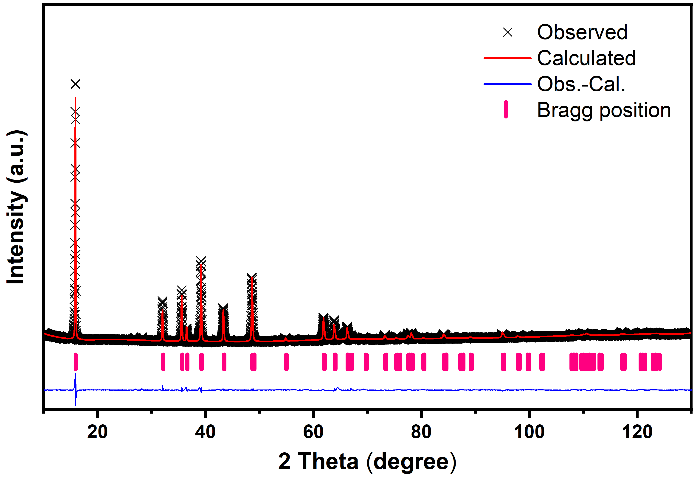


**Figure S2.** Rietveld refinement of the XRD pattern of Na_2/3_Cu_1/3_Mn_1/2_Ti_1/6_O_2_ (NCMTO) using the *P*6_3_ space group.


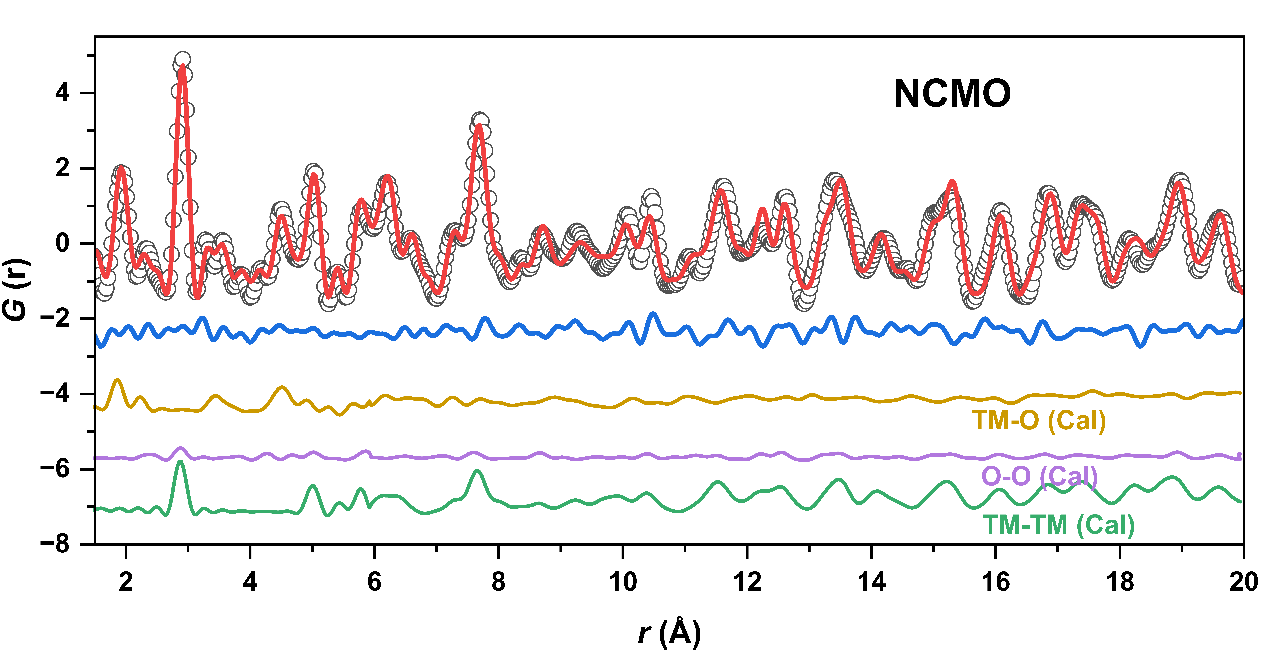


**Figure S3.** The pair distribution function (PDF) refinement of Na_2/3_Cu_1/3_Mn_2/3_O_2_ (NCMO) obtained using the *P6_3_* space group.


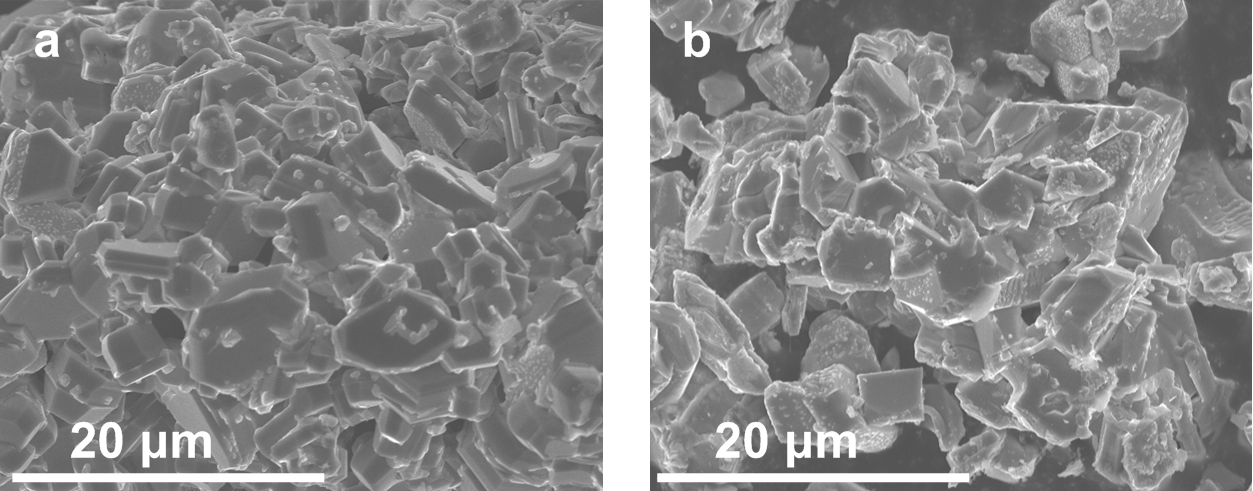


**Figure S4.** Scanning electron microscopy (SEM) images of a) NCMO and b) NCMTO.


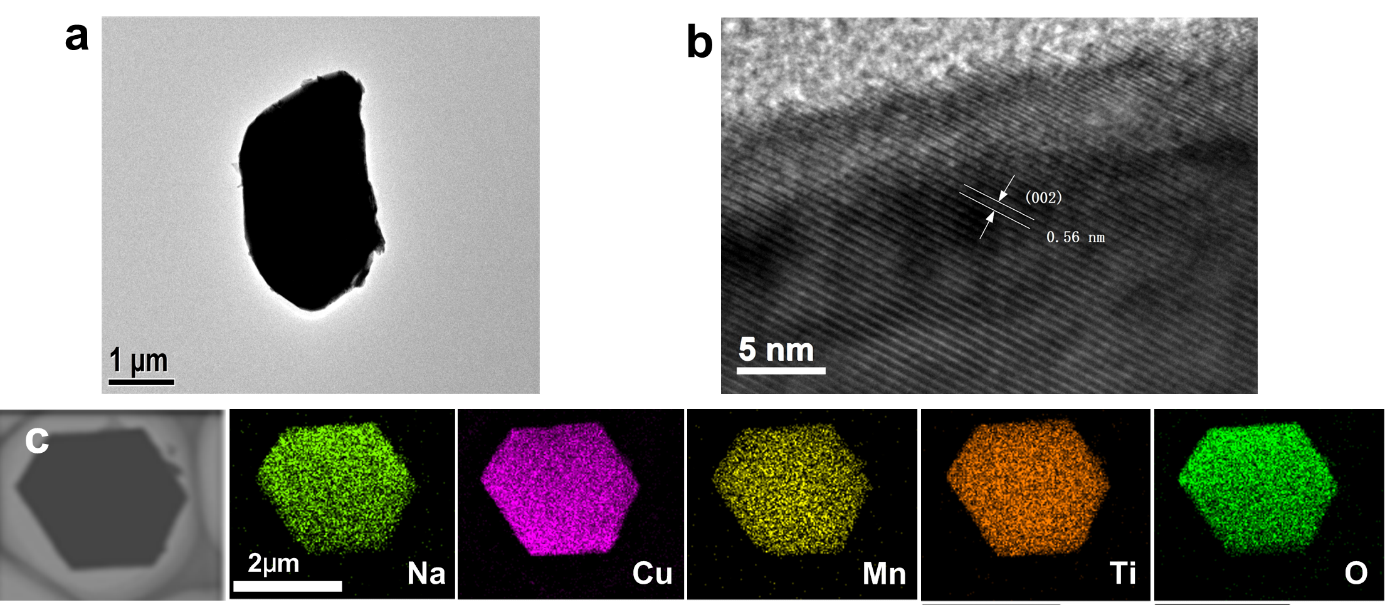


**Figure S5.** a) Transmission electron microscopy (TEM) image, b) high-resolution TEM image, and c) elemental mappings of NCMTO particle.


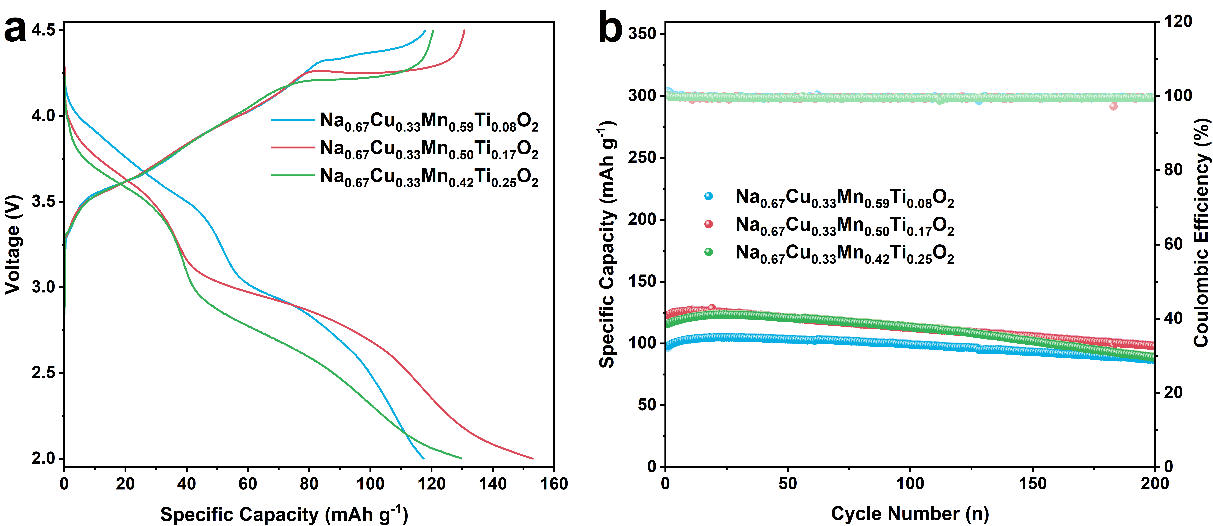


**Figure S6.** a) Initial charge–discharge curves at 0.1 C and b) cycling performance comparison at 1 C in the voltage range of 2.0-4.5 V for different Na_0.67_Cu_0.33_Mn_0.67-x_Ti_x_O_2_.


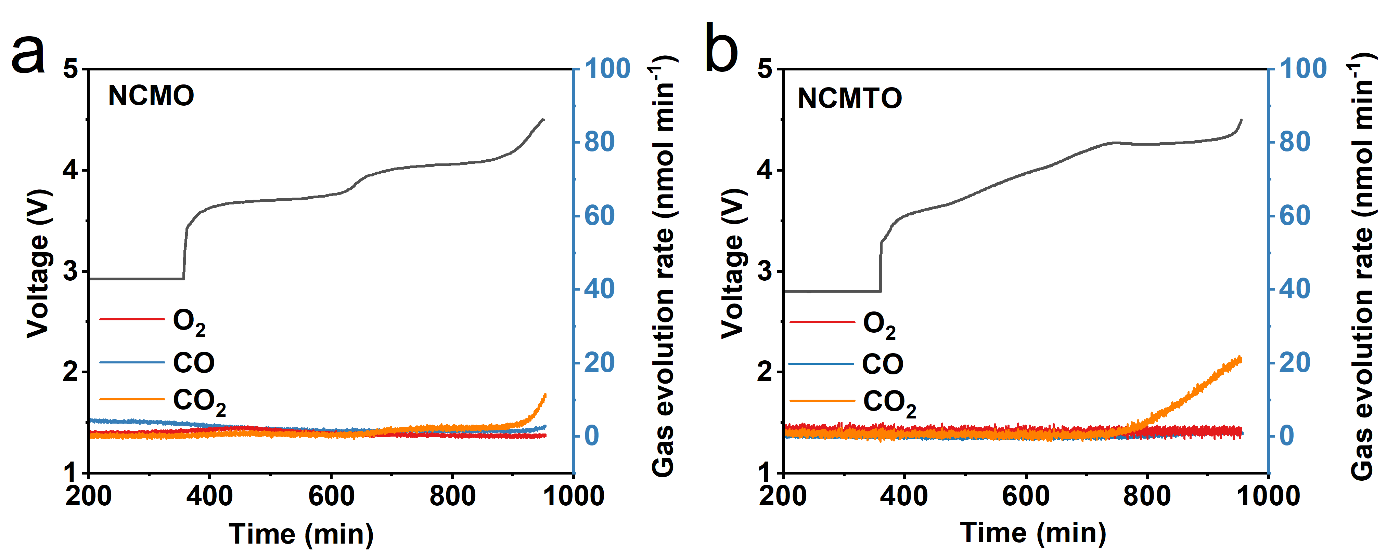


**Figure S7.** Operando differential electrochemical mass spectrometry test of a) NCMO and b) NCMTO.


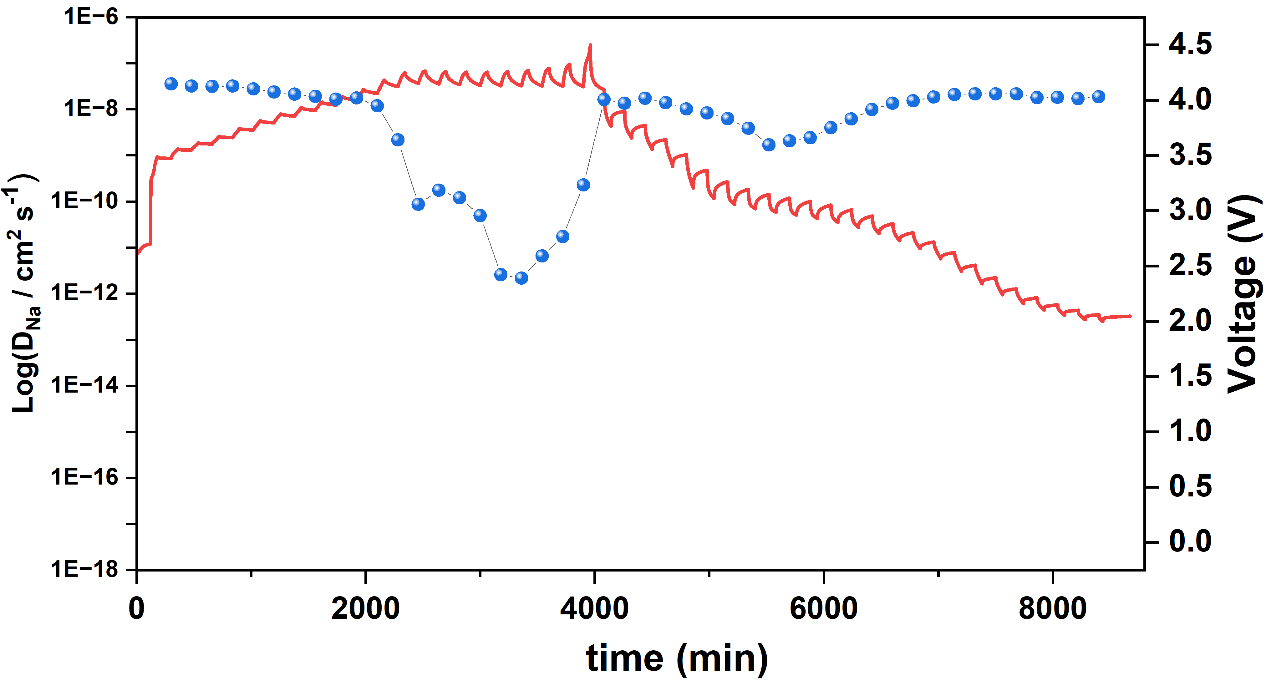


**Figure S8.** Galvanostatic intermittent titration technique (GITT) curves of NCMTO and the obtained Na diffusion coefficients.


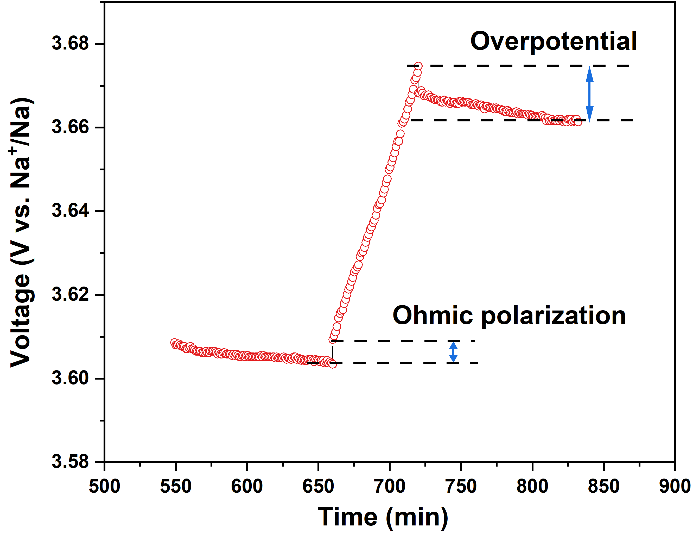


**Figure S9.** Selected steps of the GITT curves of NCMTO obtained during charging.

The chemical diffusion coefficient of Na (*D*_Na_) was calculated from galvanostatic intermittent titration technique (GITT) data using Equation (1).^[7]^

$D_{Na}=\frac{4}{\pi\tau}\left( \frac{m_{B}V_{m}}{M_{B}S} \right)^{2}\left( \frac{\Delta E_{S}}{\Delta E_{\tau}} \right)^{2}\left( \tau\leq\frac{L^{2}}{D_{{Na}^{+}}} \right),$ (1)

where *τ* (s) is the duration of the current pulse, *m*_B_ (g) and *M*_B_ (g mol^−1^) are the mass and molecular weight of electrode material, respectively, *V*_m_ (cm^3^ mol^−1^) is the molar volume of electrode material, *S* is the electrode surface area, and Δ*E*_τ_ (V) and Δ*E*_s_ (V) are the transient voltage change during the current pulse and steady voltage change after the relaxation period, respectively, *L* (cm) is the characteristic length of electrode material.


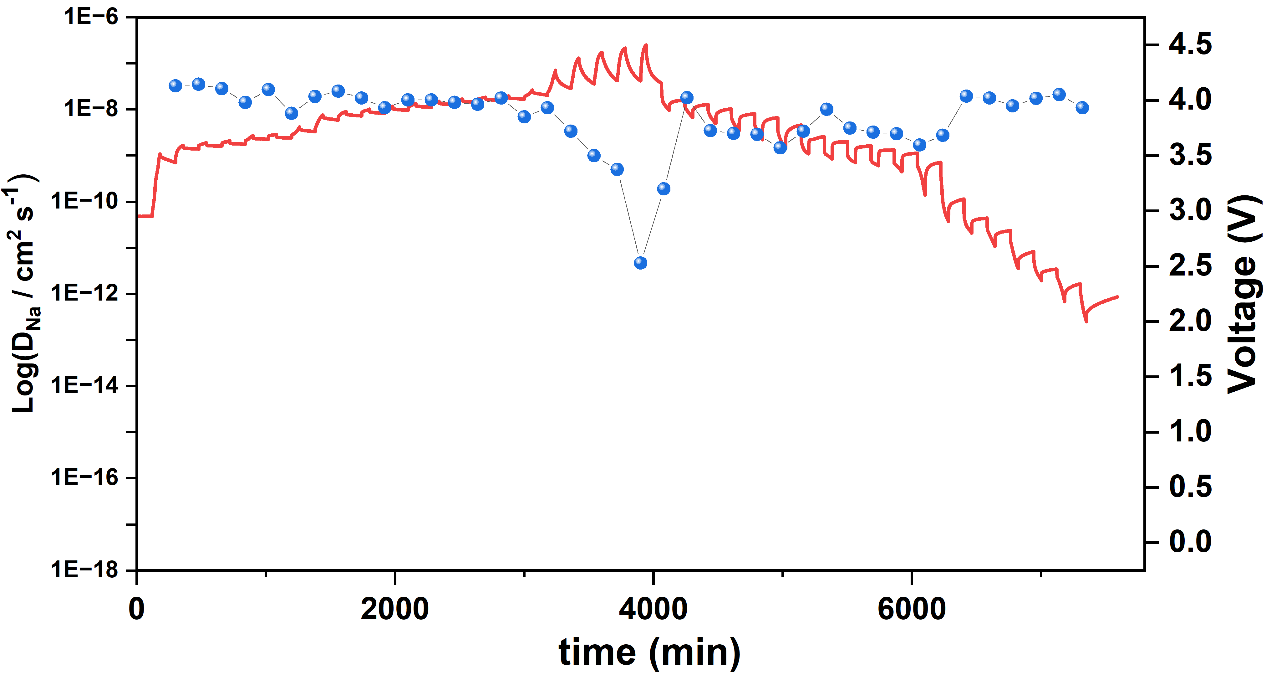


**Figure S10.** GITT curves of NCMO and the obtained Na diffusion coefficients.


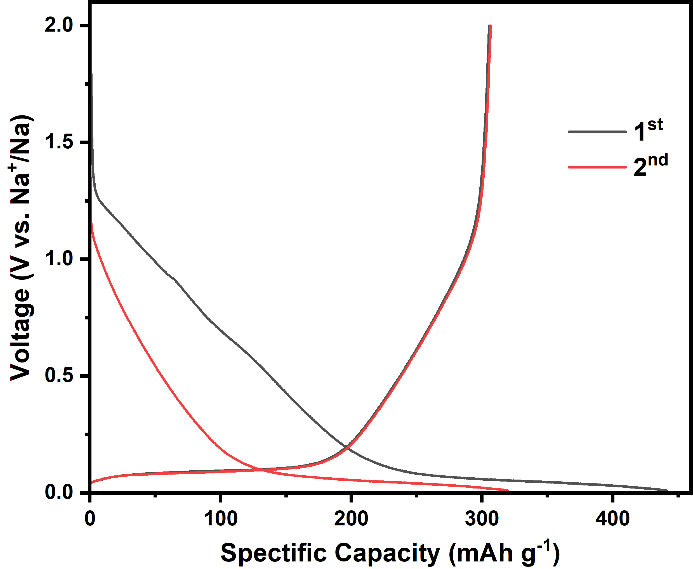


**Figure S11.** First two charge–discharge curves of hard carbon.


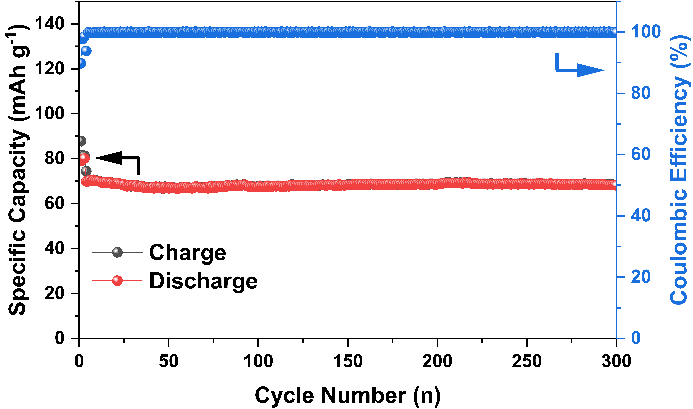


**Figure S12.** Cycling performance of NCMO at a 2 C rate.


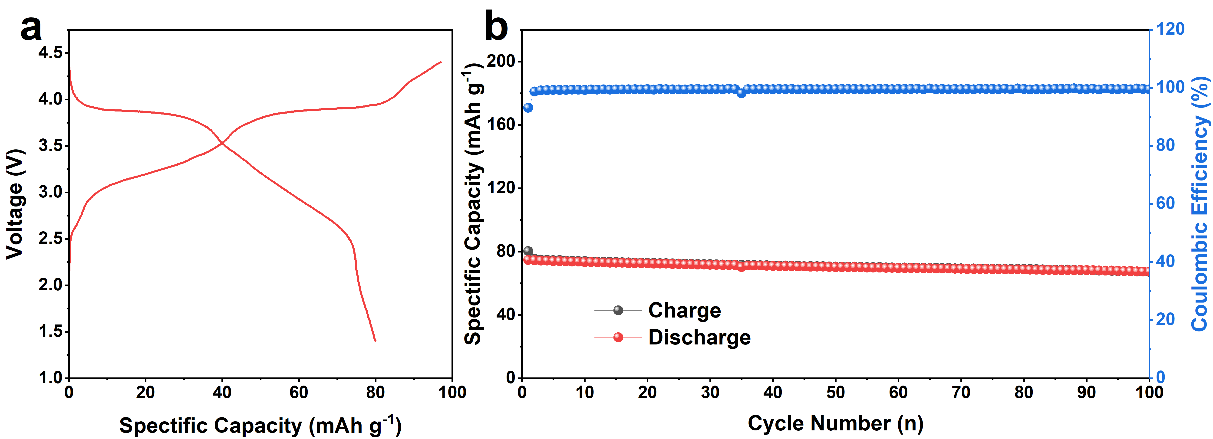
**Figure S13.** a) Initial charge–discharge curves at 0.1 C and b) cycling performance at 1 C of NCMO//hard carbon full-cells.


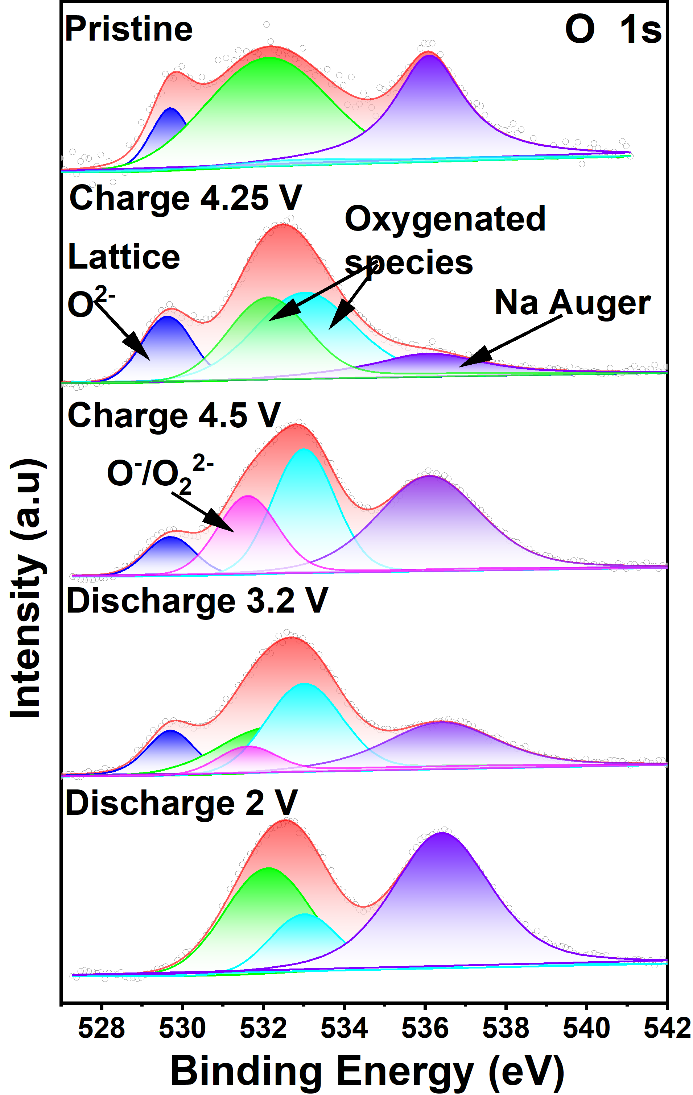


**Figure S14.** O 1s spectra of NCMTO in different charge and discharge states.


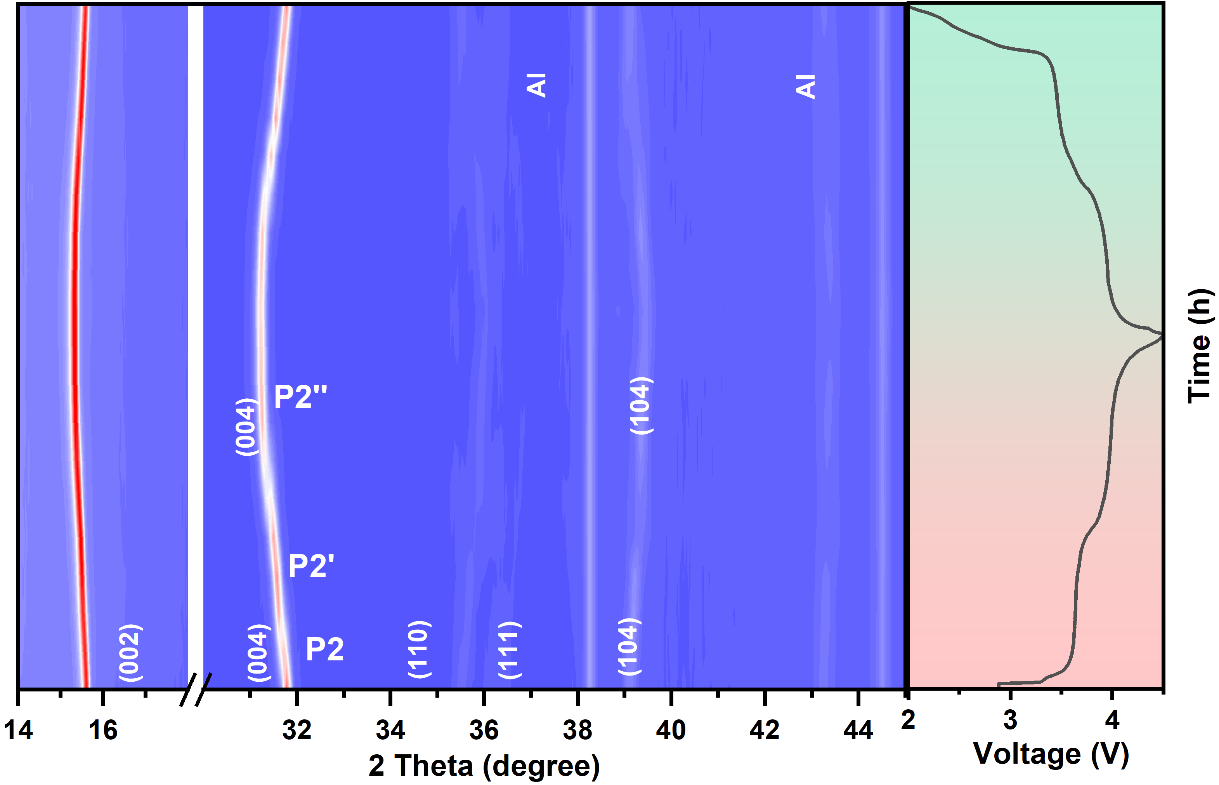


**Figure S15.** In situ XRD contour plot of NCMO obtained within the voltage range of 2–4.5 V.


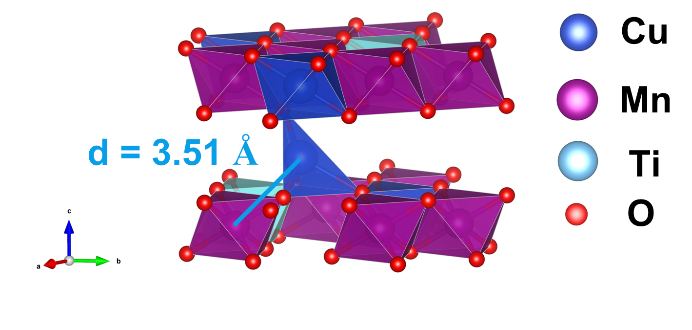


**Figure S16.** Crystal structure model used for PDF analysis.


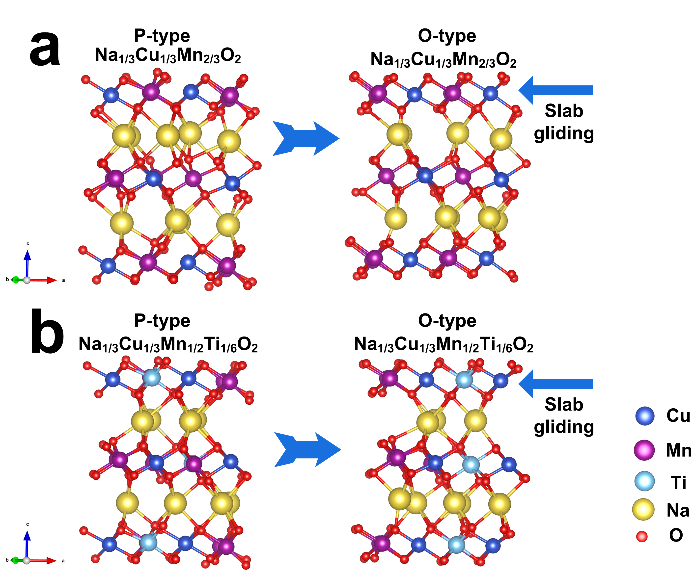


**Figure S17.** Schematic of transition metal (TM) slab gliding in a) NCMO and b) NCMTO.


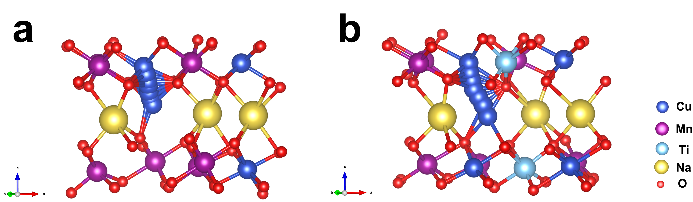


**Figure S18.** Migration paths in a) NCMO and b) NCMTO.


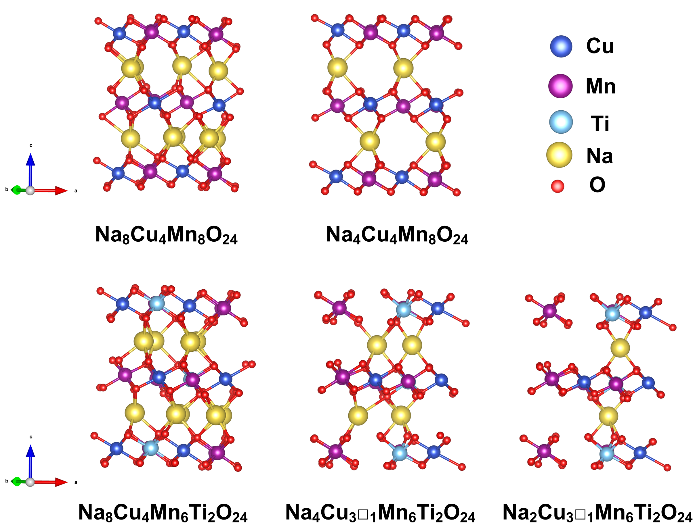


**Figure S19.** Structures of Na*_x_*Cu_4_Mn_8_O_24_ (*x* = 8 and 4) and Na_8_Cu_4_Mn_6_Ti_2_O_24_ (*x* = 8, 4, and 2). Configurations with vacancies in the TM layers of Na*_x_*Cu_3_□_1_Mn_6_Ti_2_O_24_ are derived from structures where Ti-substituted materials were used after the removal of half of the Na^+^ ions.


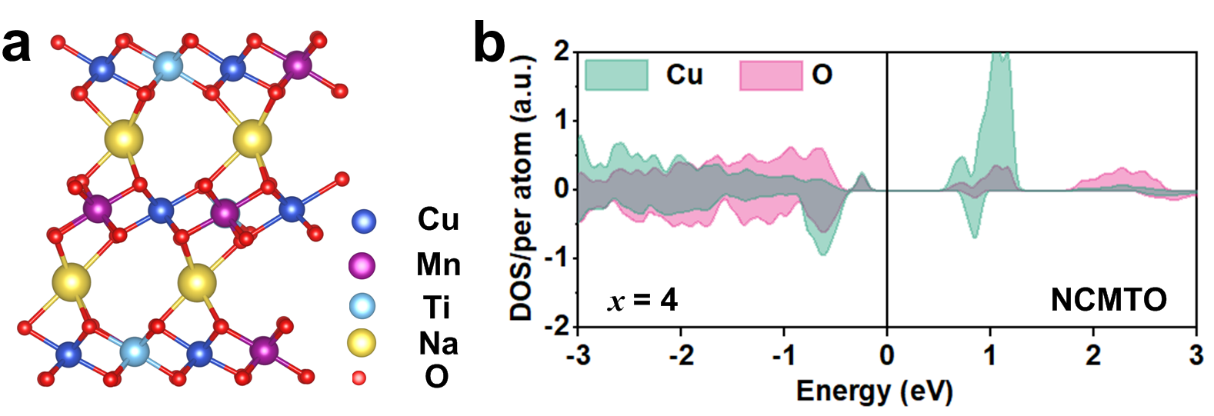


**Figure S20.** a) Schematic depicting a vacancy-free TM layer within the Na_4_Cu_4_Mn_6_Ti_2_O_24_ structure. b) Projected density of states profile of Na_4_Cu_4_Mn_6_Ti_2_O_24_.


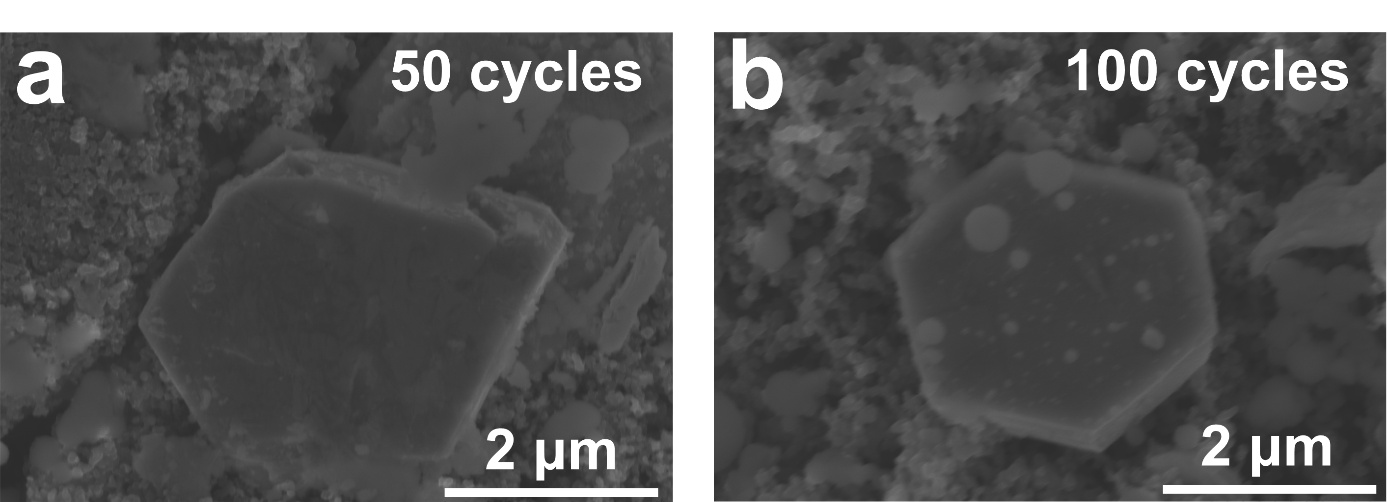


**Figure S21.** SEM images of NCMTO after 50 and b) 100 cycles.

**Table S1.** Compositions of NCMO and NCMTO determined by inductively coupled plasma-atomic emission spectrometry (ICP-AES).

| Sample | Na [wt%] | Cu [wt%] | Mn [wt%] | Ti [wt%] | Molar fraction |
| --- | --- | --- | --- | --- | --- |
| NCMO | 13.5923 | 18.7299 | 34.1511 |  | 0.645:0.322:0.678 |
| NCMTO | 13.6836 | 18.5629 | 25.6170 | 6.3459 | 0.668:0.328:0.523:0.149 |

**Table S2.** Crystallographic parameters of NCMO obtained by Rietveld refinement using the *P*6_3_ space group.

|  | Site | *x* | *y* | *z* | occupancy |
| --- | --- | --- | --- | --- | --- |
| Na1 | 6c | 0.692(0) | 0.620(0) | 0.235(0) | 0.141 |
| Na2 | 2a | 0.000(0) | 0.000(0) | 0.236(0) | 0.491 |
| Na3 | 2a | 0.333(3) | 0.666(7) | 0.234(0) | 0.156 |
| Na4 | 2a | 0.666(7) | 0.333(3) | 0.234(0) | 0.141 |
| Mn1 | 2a | 0.000(0) | 0.000(0) | 0.000(0) | 0.995 |
| Cu1 |  |  |  |  | 0.005 |
| Mn2 | 2b | 0.333(3) | 0.666(7) | 0.000(0) | 0.982 |
| Cu2 |  |  |  |  | 0.018 |
| Mn3 | 2b | 0.666(7) | 0.333(3) | 0.000(0) | 0.023 |
| Cu3 |  |  |  |  | 0.977 |
| O1 | 6c | 0.329(8) | 0.325(5) | 0.425(1) | 1.000 |
| O2 | 6c | 0.685(2) | 0.655(5) | −0.420(3) | 1.000 |
| *P*6_3_ | *a* = 5.0242(5) | *c* = 11.160(0) | *V* = 243.97(0) | *R*_p_ = 2.12% | *R*_wp_ = 4.34% |

**Table S3.** Crystallographic parameters of NCMTO obtained by Rietveld refinement using the *P*6_3_ space group.

|  | Site | *x* | *y* | *z* | occupancy |
| --- | --- | --- | --- | --- | --- |
| Na1 | 6c | 0.692(0) | 0.620(0) | 0.235(0) | 0.141 |
| Na2 | 2a | 0.000(0) | 0.000(0) | 0.236(0) | 0.491 |
| Na3 | 2a | 0.333(3) | 0.666(7) | 0.234(0) | 0.152 |
| Na4 | 2a | 0.666(7) | 0.333(3) | 0.234(0) | 0.146 |
| Mn1 | 2a | 0.000(0) | 0.000(0) | 0.000(0) | 0.654 |
| Cu1 |  |  |  |  | 0.130 |
| Ti1 |  |  |  |  | 0.216 |
| Mn2 | 2b | 0.333(3) | 0.666(7) | 0.000(0) | 0.706 |
| Cu2 |  |  |  |  | 0.106 |
| Ti2 |  |  |  |  | 0.189 |
| Mn3 | 2b | 0.666(7) | 0.333(3) | 0.000(0) | 0.141 |
| Cu3 |  |  |  |  | 0.764 |
| Ti3 |  |  |  |  | 0.095 |
| O1 | 6c | 0.326(0) | 0.306(8) | 0.410(3) | 1.000 |
| O2 | 6c | 0.680(6) | 0.673(0) | 0.410(3) | 1.000 |
| *P*6_3_ | *a* = 5.0505(0) | *c* = 11.1879(1) | *V* = 247.18(1) | *R*_p_ = 2.32% | *R*_wp_ = 4.35% |

**Table S4.** Crystallographic parameters of NCMTO obtained by Rietveld refinement using the *P*6_3_/*mmc* space group.

|  | Site | *x* | *y* | *z* | occupancy |
| --- | --- | --- | --- | --- | --- |
| Nae | 2d | 0.666(7) | 0.333(3) | 0.250(0) | 0.329 |
| Naf | 2b | 0.000(0) | 0.000(0) | 0.250(0) | 0.341 |
| Mn | 2a | 0.000(0) | 0.000(0) | 0.000(0) | 0.502 |
| Cu |  |  |  |  | 0.330 |
| Ti |  |  |  |  | 0.167 |
| O | 4f | 0.666(7) | 0.333(3) | 0.057(3) | 1.000 |
| *P*6_3_/*mmc* | *a* = 2.914(8) | *c* = 11.183(2) | *V* = 82.28(3) | *R*_p_ = 1.96% | *R*_wp_ = 3.52% |

**Table S5.** Performances of recently reported P-type layered oxide cathode materials with fully occupied TM layers.

| Category | Cathode material | Voltage  range | Initial capacity | Structure  change | Refs. |
| --- | --- | --- | --- | --- | --- |
| P2 | Na_2/3_Ni_1/3_Mn_2/3_O_2_ | 2.6–4.3 | 120 | P2 to O2 | ^[8]^ |
| P2 | Na_0.78_Ni_0.23_Mn_0.69_O_2_ | 2.0–4.5 | 138 | P2 to O2 | ^[9]^ |
| P2 | Na_0.78_Co_1/2_Mn_1/3_Ni_1/3_O_2_ | 2.0–4.5 | 146 | P2 to O2 | ^[10]^ |
| P3 | Na_0.5_Ni_0.25_Mn_0.75_O_2_ | 3.75–4.25 | 180 | P3 to O3 | ^[11]^ |
| P2 | Na_0.67_Mn_0.66_Fe_0.20_Cu_0.14_O_2_ | 1.5–4.3 | 176 | P2 to Z | ^[12]^ |
| P2 | Na_0.67_Fe_0.2_Mn_0.65_Ni_0.15_O_2_ | 1.5–4.3 | 200 | P2 to Z | ^[13]^ |
| P2 | Na_0.67_Fe_1/2_Mn_1/2_O_2_ | 1.5–4.2 | 190 | P2 to OP4 | ^[14]^ |
| P’2 | Na_0.67_[(Mn_0.78_Fe_0.22_)_0.9_Ti_0.1_]O_2_ | 1.5–4.3 | 180 | P′2 to OP4 | ^[15]^ |
| P’2 | Na_0.67_Ni_0.1_Fe_0.1_Mn_0.8_O_2_ | 1.5–4.3 | 220 | P′2 to OP4 | ^[16]^ |

**References**

[1] N. Su, Y. Lyu, B. Guo, *Electrochem. Commun.* **2018**, *87*, 71.

[2] G. Kresse, D. Joubert, *Phys. Rev. B* **1999**, *59*, 1758.

[3] J. P. Perdew, K. Burke, M. Ernzerhof, *Phys. Rev. Lett.* **1996**, *77*, 3865.

[4] S. Grimme, J. Antony, S. Ehrlich, H. Krieg, *J. Chem. Phys.* **2010**, *132*, 154104.

[5] D. Wines, K. Saritas, C. Ataca, *J. Phys. Chem. C* **2022**, *126*, 5813.

[6] L. I. Bendavid, E. A. Carter, *J. Phys. Chem. C* **2013**, *117*, 26048.

[7] W. Weppner, R. A. Huggins, *J. Electrochem. Soc.* **1977**, *124*, 1569.

[8] Y. Zhang, M. Wu, J. Ma, G. Wei, Y. Ling, R. Zhang, Y. Huang, *ACS Cent. Sci.* **2020**, *6*, 232.

[9] C. Ma, J. Alvarado, J. Xu, R. J. Clément, M. Kodur, W. Tong, C. P. Grey, Y. S. Meng, *J. Am. Chem. Soc.* **2017**, *139*, 4835.

[10] C. Hakim, N. Sabi, L. A. Ma, M. Dahbi, D. Brandell, K. Edström, L. C. Duda, I. Saadoune, R. Younesi, *Commun. Chem.* **2020**, *3*, 9.

[11] Q. Li, Y. Qiao, S. Guo, K. Jiang, Q. Li, J. Wu, H. Zhou, *Joule* **2018**, *2*, 1134.

[12] E. Talaie, S. Y. Kim, N. Chen, L. F. Nazar, *Chem. Mater.* **2017**, *29*, 6684.

[13] E. Talaie, V. Duffort, H. L. Smith, B. Fultz, L. F. Nazar, *Energy Environ. Sci.* **2015**, *8*, 2512.

[14] N. Yabuuchi, M. Kajiyama, J. Iwatate, H. Nishikawa, S. Hitomi, R. Okuyama, R. Usui, Y. Yamada, S. Komaba, *Nat. Mater.* **2012**, *11*, 512.

[15] Y. J. Park, J. U. Choi, J. H. Jo, C.-H. Jo, J. Kim, S.-T. Myung, *Adv. Funct. Mater.* **2019**, *29*, 1901912.

[16] J. U. Choi, J. H. Jo, Y. J. Park, K.-S. Lee, S.-T. Myung, *Adv. Energy Mater.* **2020**, *10*, 2001346.
